# Supplementary material for: Magnetic-Field-Assisted Molecular Beam Epitaxy: Engineering of Fe3O4 Ultrathin Films on MgO(111)
Source: Materials (Basel). 2023 Feb 10;16(4):1485. doi: 10.3390/ma16041485 (PMC9964408; doi:10.3390/ma16041485)
Supplement: Supplementary file 1 [file materials-16-01485-s001.zip › materials-2186491-supplementary.pdf]

Supplementary Material

Magnetic-Field-Assisted Molecular Beam Epitaxy: Engineering of Fe_3O_4 Ultrathin Films on MgO(111)

Adam Dziwoki [1,2], Bohdana Blyzniuk [1], Kinga Freindl [1], Ewa Madej [1], Ewa Młyńczak [1], Dorota Wilgocka-Ślęzak [1], Józef Korecki [1,*] and Nika Spiridis [1]

[1] Jerzy Haber Institute of Catalysis and Surface Chemistry, Polish Academy of Sciences, Niezapominajek 8, 30-239 Krakow, Poland
[2] PREVAC sp. z o.o., Raciborska Str. 61, 44-362 Rogów, Poland
* Correspondence: jozef.korecki@ikifp.edu.pl

Table S1. Best fit parameters of the CEMS spectra octahedral (A) and tetrahedral (B) sites. B_{hf} is hyperfine magnetic field, IS is isomer shift relative to α-Fe, ε is quadrupole shift.

Sample	Site	B_{hf} [T]	IS [mm/s]	ε [mm/s]	Relative intensity %
No-field	A	47.5 (1)	0.26(1)	0.00(1)	33(1)
	B	44.9 (2)	0.63(1)	0.00(1)	67(1)
In-field	A	47.4 (2)	0.26(1)	0.01(1)	32(1)
	B	44.7(2)	0.63(1)	0.00(1)	68(1)

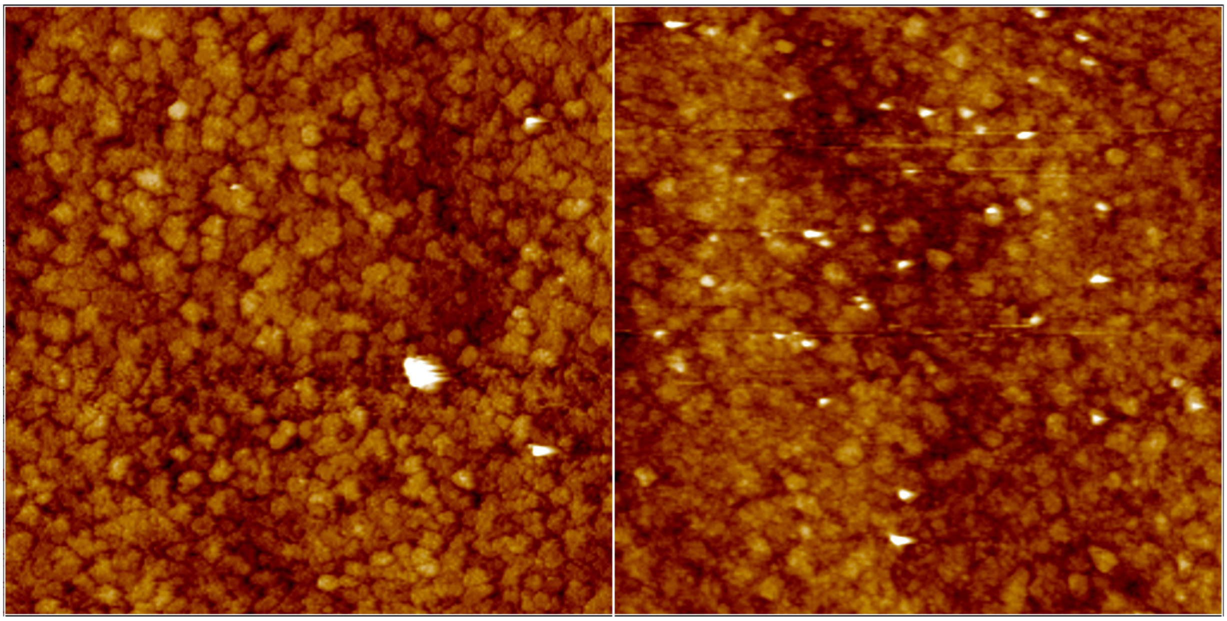

Figure S1 1x1 μm^2 STM images for the in-field and no-field samples, left and right, respectively.

Simulations of the P-MOKE loops

Simulations that best reproduced the measured hard P-MOKE loops were performed by minimizing the total energy in the form:

$$E(\theta,\varphi) = -\frac{\sqrt{3}}{3} H\, M_s\, [\sin\theta\cos\varphi + \sin\theta\ \sin\varphi + \cos\theta] + K_1[sin^4\theta\ \ sin^2\varphi\, cos^2\varphi + sin^2\theta\ \ cos^2\theta] + K_2[sin^4\theta\ \ cos^2\theta\, sin^2\varphi\, cos^2\varphi] + (2\pi M_s^2 - K_u)[\frac{1}{3} + \frac{2}{3} sin^2\theta\ \ \sin\varphi\cos\varphi + \frac{2}{3}\sin\theta\ cos\theta\ (\sin\varphi + \cos\varphi)] + K_u\gamma[\frac{1}{3} + \frac{2}{3} sin^2\theta\ \ \sin\varphi\cos\varphi + \frac{2}{3}\sin\theta\ cos\theta\ (\sin\varphi + \cos\varphi)]^2 .$$

θ and φ are polar and azimuthal angles in the Cartesian coordinate system associated with the cubic unit cell of magnetite, and the formula above takes into account the (111)-orientation of the magnetite film and the magnetic field H that is applied along the film normal. M_s is the bulk magnetite magnetization and K_1 and K_2 are first- and second-order bulk magnetocrystalline anisotropy constants. The fourth and fifth term of the formula express the film-typical contributions to the total energy: the shape anisotropy that scales with $2\pi M_s^2$, and an additional phenomenologically introduced uniaxial anisotropy constants of the first- (K_u) and second- ($K_u\gamma$) order, which are necessary to counterbalance and overwhelm the shape anisotropy and stabilize the out-of-plane magnetization component. We are aware that such description is an oversimplification of a complex magnetization structure caused by the presence of the antiphase boundaries (compare Ref. 40 in the main text), but it allows quantification of the anisotropy changes induced by the in-field deposition.

From the equilibrium magnetization direction derived with the equilibrium conditions: $\frac{\partial E}{\partial \theta} = \frac{\partial E}{\partial \varphi} = 0$, the out-of-plane magnetization curves are plotted in Fig. 1S and 2S for K_u and γ that give the best simulation of the experimental data obtained for the no-field and in-field samples, respectively. Note that the simulations in the single-domain model are not able to properly reproduce the hysteresis. The derived K_u and γ values are given in the figure captions.

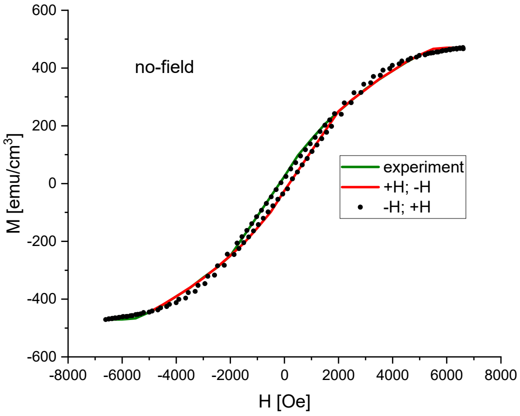

Figure S2 $K_u = 7.7*10^5$ erg/cm^3 , $\gamma = 0.48$

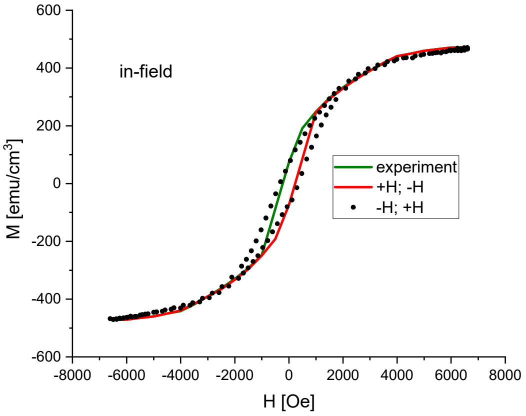

Figure S3 $K_u = 12.8*10^5$ erg/cm^3 , $\gamma = 0.42$
